# Human T-lymphotropic Virus Type I (HTLV-I) Proviral Load and Clinical Features in Iranian HAM/TSP Patients*: Comparison of HTLV-I Proviral Load in HAM/TSP Patients*

**Published:** 2013-03

**Authors:** Rosita Vakili, Faezeh Sabet, Sanaz Aahmadi, Reza Boostani, Houshang Rafatpanah, Ali Shamsian, S. A. Rahim Rezaee

**Affiliations:** 1 HTLV-I Foundation, Ghaem Hospital, Faculty of Medicine, Mashhad University of Medical Sciences, Mashhad, Iran; 2 Centre of Pathological and Medical Diagnostic Services, Iranian Academic Centre for Education, Culture & Research (ACECR), Mashhad Branch, Mashhad, Iran; 3 Department of Neurology, Ghaem Hospital, Faculty of Medicine, Mashhad University of Medical Sciences, Mashhad, Iran; 4 Inflammation and Inflammatory Diseases Research Centre, School of Medicine, Mashhad University of Medical Sciences, Mashhad, Iran; 5 Immunology Research Centre, School of Medicine, Mashhad University of Medical Sciences, Mashhad, Iran

**Keywords:** HTLV-I, HAM/TSP, HTLV-I clinical features, Iran, Proviral load

## Introduction

Human T-cell lymphotropic virus type I (HTLV-I) was the first human retrovirus discovered (1), and it has been estimated that 10-20 million people worldwide are infected with HTLV-I (2). This virus is endemic in several regions of the world, such as southwestern Japan, the Caribbean basin, Central Africa, South America, the Melanesian Islands, and the Middle East (3, 4). The prevalence of HTLV-I infection in Iran (Mashhad) is estimated to be 2-3% of the entire population, and 0.7% among blood donors (5). Most of HTLV-I-infected individuals remain asymptomatic carriers (6). Whereas, small percentage of infected individuals develop the neoplastic disease adult T-cell leukemia (ATL), and the inflammatory condition HTLV-I- associated myelopathy/tropical spastic paraparesis (HAM/TSP) (7). Only 5% of HTLV-I infected people develop HAM/TSP (8).

HAM/TSP results in demyelination of the spinal cord and clinical manifestations of this disease include progressive muscle weakness and hyperreflexia of the lower limbs, sensory disturbance, urinary incontinence, and impotence (9-11). These symptoms are generally slowly progressive, while patients at older ages of onset show faster progression. Women are affected more frequently than men (12, 13).

The precise pathophysiology of HAM/TSP is not yet clear but, previous studies suggested that both HTLV-I subgroups, host genetic and immunological factors may be associated with the development of HAM/TSP, particularly cytokine gene polymorphisms (14, 15). Proviral load is a major determinant of outcome for chronic virus infections such as HIV-1 and 2, hepatitis B virus, and hepatitis C virus. Also recent studies have suggested the important role of proviral load in the outcome of human T-lymphotropic virus-I infection (16).

In patients with HAM/TSP, significantly higher proviral loads have been observed compared to asymptomatic carriers, suggesting that active HTLV-I viral replication plays a key role in the development of this disease. A recent study has shown that a high level of Tax expression and low CD8+ anti-viral efficiency are correlated with high proviral load (PVL) and HAM/TSP development (17).

In a previous study the proviral load and host genetic risk factors for development of HAM/TSP between Iranian and Japanese HTLV-I infected individuals were compared, and it was found that the median HTLV-I proviral load of Iranian HAM/TSP patients was two-fold greater in HAM/TSP patients than in healthy carriers (HCs), whereas that of Japanese HAM/TSP patients was 13-fold greater than in HCs. In addition, The HTLV-I proviral load in Iranian HCs was significantly higher than Japanese HCs. These significant differences of proviral load between two populations reflect the role of genetic factors such as the human leukocyte antigen (HLA) genotype in populations. In this study the Iranian genome DNA samples were extracted from the whole blood but Japanese samples were from the peripheral blood mononuclear cells (PBMCs). The Iranian proviral load was probably underestimated in comparison with Japanese ones (18). The aim of this study was to evaluate the proviral load and clinical manifestation of HAM/TSP among Iranian to compare with other endemic parts of the world.

## Materials and Methods

*Study population*

In this retrospective study, files of 102 HAM/TSP patients and 34 HCs from Iranian individuals whom were referred to ACECR Khorasan Central Medical Lab and Navid Medical Lab (Mashhad, Iran) were reviewed. All these subjects were resident of Khorasan province, Iran. Japanese study population consisted of 222 HAM/TSP patients and 184 HCs from Kogoshima, Japan and Brazilian population included 92 HAM/TSP patients and 242 HCs from Salvador, Brazil. HTLV-I infection was determined using a HTLV-I antibody serological test. The diagnosis of HAM/TSP was performed in accordance with the World Health Organization criteria. The demographic information (including age, gender, and duration of disease), and neurological symptoms of HAM/TSP were collected from medical recorded files in Ghaem Hospital, Mashhad University of Medical Sciences (Mashhad, Iran). HTLV-I proviral load and clinical manifestation in Japanese and Brazilian populations obtained from published studies (14, 11, 18-23).

*HTLV-I proviral load measurement*

To examine the HTLV-I proviral load, peripheral blood mononuclear cells (PBMCs) were isolated from EDTA-treated blood samples by Ficoll density gradient (Sigma, Germany). A real time PCR using a commercial absolute quantification kit (Novin Gene, Iran) was performed to measure the proviral load of HTLV-I in PBMCs using specific primers, and a fluorogenic probe by a Rotorgen Q (Qiagen, Germany) Real-Time PCR machine. The HTLV-I copy number was reported as an actual amount of cellular DNA by means of quantification of the albumin gene as the reference gene. HTLV-I and albumin DNA concentrations were calculated from two 5-point standard curves. The normalized value of the HTLV- I proviral load was calculated as the ratio of (HTLV-I DNA copies number/albumin DNA copies number/2)×10^4^ and expressed as the number of HTLV-I proviruses per 10^4^ PBMCs (24).

*Statistical analysis*

Data was analyzed by Nonparametric Mann-Whitney and Spearman’s correlation tests using SPSS/ver.19 software. Descriptive data were summarized as mean, standard error (SE), and percents. A p value < 0.05 was considered as statistically significant, and 95% confidence intervals (CI) were also estimated.

## Results

*HTLV-I proviral load*

HTLV-I proviral load was determined in 136 Iranian individuals infected with HTLV-I. Of them, 102 subjects were HAM/TSP patients and 34 subjects were HCs. Eighty nine of them (65.4%) were females and forty seven (34.6%) were males. The mean age of them was 42.22 ± 1.250 years (range 11-79 years). Maximum HTLV-I proviral load was 2578 copy number/10^4^ PBMCs in an immune-compromised patient, and minimum HTLV-I proviral load was 10 copy number/10^4^ PBMCs in a HCs subject. The mean age of HAM/TSP patients (43.07 ± 1.478 years) was higher than HCs group (39.68 ± 2.290 years). Characteristics and proviral load of HAM/TSP patients and HCs subjects are summarized in Table 1. Table 2 shows the characteristics and HTLV-I proviral load in HAM/TSP patients and HCs subjects based on gender. HTLV-I proviral load in HAM/TSP patients and HCs was not significantly associated with age and gender.

*Clinical manifestations*

The most common HAM/TSP neurological symptoms among Japanese were gait impairment (65%), urinary disturbance (33%), numbness of lower legs (13%), constipation (6%), and lumbago (9%) (19).The time between infection with HTLV-I virus and the onset of HAM/TSP usually varies from months to decades (20). In Iranian HAM/TSP patients the most common HTLV-I related manifestations were gait impairment (95%), urinary disturbance (93%), fatigue, and weakness (85%), paresthesias (78.2%), constipation (75%), and pain (73%). The most common clinical features of HAM/TSP patients in Brazilian population were pyramidal syndrome in lower limbs (100%), motor disability (73%), low back pain (62.5%), sensory deficits (52.3%), hand numbness (35%), increased tendon jerks in upper limbs (28.4%), foot numbness (23.9%), and sphincter problems (11%). According to our studies most of the HAM/TSP patients in northeast of Iran were observed in Mashhad (57.27%), Neishabour, and then Quchan. However the highest rates of HAM/TSP have been seen in Neishabour. The onset of HAM/TSP in this region seems to be around 10 years.

**Table 1:** Characteristics and HTLV-I Proviral Load of HAM/TSP patients and Healthy Carrier Subjects

Characteristics	Group	
	HAM/TSP	Healthy carrier
No. subjects (%)	102(75)	34(25)
Proviral load (mean ± SE) HTLV-I copy number/104 PBMCs	626.16±53.031	193±44.375
Minimum proviral load	12	10
Maximum proviral load	2578	1171
CI	520.96-731.36	102.72-283.28
Age (mean years)	43.07±1.478	39.68±2.290

**CI; confidence interval, SE; standard error**

**Table 2:** Characteristics and HTLV-I Proviral Load of HAM/TSP Patients and Healthy Carrier Subjects Based on Gender

Characteristics	Group			
	HAM/TSP		Healthy carrier	
Gender	Female	Male	Female	Male
No. subjects (%)	69(67.6)	33(32.4)	20(58.8)	14(41.2)
Proviral load (mean ± SE) HTLV-I copy number/104 PBMCs	548.4±54.15	789.48±114.76	142.50±47.93	265.14±81.61
Minimum proviral load	12	58	10	20
Maximum proviral load	2453	2578	980	1171
CI	439.97-656.12	555.72-1023.25	42.17-242.83	88.82-441.47
Age (mean years)	42.54±1.571	44.18±3.205	36.10±3.076	44.79±3.024

**CI; confidence interval, SE; standard error**

## Discussion

Previous studies have demonstrated that host genetics, together with viral factors, are associated with an increased risk of developing HAM/TSP, and clinical progression of this disease. The most important factors are HTLV-I proviral load, HTLV-I subgroups, HLA background, frequency of HTLV-I-specific CD4^+^ T cells, age, gender, routes of transmission (i.e., breastfeeding or transfusion), and high antibody titers (25-29).

In this study, HTLV-I proviral load and clinical manifestation of HAM/TSP among Iranian HTLV-I infected individuals have been evaluated, and were compared to the results of other endemic parts of the world.

The median age of HAM/TSP patients in Iranian (43 years, range 11–79 years), Japanese (57.3 years, range 15–80 years) and Brazilian populations (54 years, range 31-73 years) were higher than Iranian (35.50 years, range 19-68 years), Japanese (39.4 years, range 16–64 years), and Brazilian (38 years, range 15-74 years) HCs (14, 18). These results are consistent with other studies (16, 30). Also the mean age of females having HAM/TSP was higher than males having HAM/TSP in Iranian individuals (Table 2).

As expected, the mean HTLV-I proviral load of HAM/TSP patients in Iranian (626.16 ± 53.031copies/10^4 ^PBMCs), Japanese (798 ± 51copies/10^4^PBMCs) and Brazilians (912.5 ± 778.6 copies/10^4 ^PBMCs) were greater than healthy carriers (Iranian; 193 ± 44.375, Japanese; 120 ± 17 and Brazilians, 240.5 ± 452.8 copies/10^4^PBMCs) (21-23). Mean proviral load of Iranian males in both HAM/TSP and HCs groups was higher than females. In contrast, mean proviral load of Japanese females was higher than males (21). In another cohort study conducted in 2010, mean proviral load of Japanese females was lower than males (31). These inconsistencies between the results might be due to the different methodology or distinct studies population. The mean HTLV-I proviral loads of Japanese and Brazilian HAM/TSP patients were greater than the mean HTLV-I proviral load of Iranian HAM/TSP patients. On the other hand, the mean HTLV-I proviral load of Iranian HCs was higher than that of Japanese healthy carriers and lower than Brazilians (21-23). Another study was performed in Japan, demonstrated the same results (32). Furthermore, the median HTLV-I proviral load of Iranian HAM/TSP patients was 3 times higher than HCs individuals. In contrast, the median HTLV-I proviral load of Japanese HAM/TSP patients was 16 times greater than HCs individuals (21). These differences could be due to many factors such as host genetic, immunological factors, host-virus interactions, milieu, and socioeconomic situation among Iranian, and Japanese population.

High HTLV-I proviral load is associated with clinical progression in HAM/TSP (6); however, the clinical manifestations and the percentage of occurrences vary among different infected populations (33-35). Common clinical manifestation of HAM/TSP in Japanese have been reported as gait impairment (65%), urinary disturbance (33%), numbness of lower legs (13%), constipation (6%), and lumbago (9%) (19). Furthermore, in Brazilians have been described as lower limbs (100%), motor disability (73%), low back pain (62.5%), sensory deficits (52.3%), hand numbness (35%), increased tendon jerks in upper limbs (28.4%), foot numbness (23.9%), and sphincter problems (11%) (22,23). In contrast, in our studies among Iranian HAM/TSP patients were gait impairment (95%), urinary disturbance (93%), fatigue and weakness (85%), paresthesia (78.2%), constipation (75%), and pain (73%). Although, mean viral load and the maximum load in Iranian population have been less than other populations, the rate of HAM/TSP occurrence among HTLV-I infected subjects seems to be the same (around 3-5% of infected subjects). Taken together, it is more likely that host and environmental factors should be more effective in HAM/TSP occurrence than what we expected.

## Conclusion

Several host genetic and viral factors have been identified for developing HAM/TSP. The results of this study demonstrated that different HTLV-I proviral load among Iranian and other endemic parts of the world might be also related to milieu and host genetic background. Although, high proviral load is associated with an increased risk of developing HAM/TSP in carriers, and consequently, clinical progression of disease, lower proviral load in Iranian population seems to have the same result for HAM/TSP occurrence. Therefore, other effective factors must be involved in HAM/TSP progression, and further studies are required to clarify other unknown risk factors involved in development of HTLV-I associated diseases. Such studies can help for opening a new insight toward understanding host, microbe, and environmental interaction in pathogenesis of the viral associated diseases for a better treatment.
